# Association of Antibiotic Receipt With Survival Among Patients With Metastatic Pancreatic Ductal Adenocarcinoma Receiving Chemotherapy

**DOI:** 10.1001/jamanetworkopen.2023.4254

**Published:** 2023-03-23

**Authors:** Daniel J. Fulop, Haley M. Zylberberg, Y. Linda Wu, Anne Aronson, Arielle J. Labiner, Juan Wisnivesky, Deirdre J. Cohen, Keith M. Sigel, Aimee L. Lucas

**Affiliations:** 1 Henry D. Janowitz Division of Gastroenterology, Icahn School of Medicine at Mount Sinai, New York, New York; 2 Division of Digestive and Liver Disease, Department of Medicine, Columbia University Irving Medical Center, New York, New York; 3 Tisch Cancer Institute, Icahn School of Medicine at Mount Sinai, New York, New York; 4 Division of General Internal Medicine, Icahn School of Medicine at Mount Sinai, New York, New York

## Abstract

**Importance:**

The prognosis for patients with metastatic pancreatic ductal adenocarcinoma (PDAC) is dismal, due in part to chemoresistance. Bacteria-mediated mechanisms of chemoresistance suggest a potential role for antibiotics in modulating response to chemotherapy.

**Objective:**

To evaluate whether use of peritreatment antibiotics is associated with survival among patients with metastatic PDAC treated with first-line gemcitabine or fluorouracil chemotherapy.

**Design, Setting, and Participants:**

Using the population-based Surveillance, Epidemiology, and End Results–Medicare linked database, this retrospective cohort study analyzed data for patients diagnosed with PDAC between January 1, 2007, and December 31, 2017. Data analysis was conducted between September 1, 2021, and January 15, 2023. The population-based sample included 3850 patients with primary metastatic PDAC treated with first-line gemcitabine or fluorouracil chemotherapy. Patients who received antibiotics were matched based on propensity scores to patients who did not receive antibiotics.

**Exposures:**

Receipt of 5 or more days of oral antibiotics or 1 injectable antibiotic in the month before or after beginning first-line chemotherapy.

**Main Outcomes and Measures:**

Overall survival and cancer-specific survival. The end of follow-up was December 31, 2019, for overall survival and December 31, 2018, for cancer-specific survival.

**Results:**

Of the 3850 patients treated with first-line gemcitabine (3150 [81.8%]) or fluorouracil (700 [18.2%]), 2178 (56.6%) received antibiotics. The mean (SD) age at diagnosis was 74.2 (5.8) years and patients were predominantly women (2102 [54.6%]), White (3396 [88.2%]), and from metropolitan areas (3393 [88.1%]) in the northeastern or western US (2952 [76.7%]). In total, 1672 propensity-matched pairs were analyzed. Antibiotic receipt was associated with an 11% improvement in overall survival (hazard ratio [HR], 0.89; 95% CI, 0.83-0.96; *P* = .003) and a 16% improvement in cancer-specific survival (HR, 0.84; 95% CI, 0.77-0.92; *P* < .001) among patients treated with gemcitabine. In contrast, there was no association between antibiotic receipt and overall survival (HR, 1.08; 95% CI, 0.90-1.29; *P* = .41) or cancer-specific survival (HR, 1.12; 95% CI, 0.90-1.36; *P* = .29) among patients treated with fluorouracil. In a subgroup of gemcitabine-treated patients who received antibiotics, nonpenicillin β-lactams were associated with an 11% survival benefit (HR, 0.89; 95% CI, 0.81-0.97; *P* = .01).

**Conclusions and Relevance:**

In this cohort study, receipt of perichemotherapy antibiotics was associated with improved survival among patients treated with gemcitabine, but not fluorouracil, suggesting that antibiotics may modulate bacteria-mediated gemcitabine resistance and have the potential to improve PDAC outcomes.

## Key Points

**Question:**

Is antibiotic therapy associated with response to chemotherapy in patients with metastatic pancreatic ductal adenocarcinoma (PDAC)?

**Findings:**

In this cohort study of 3850 older adults with metastatic PDAC, receipt of perichemotherapy antibiotics was associated with an 11% improvement in survival for patients who received gemcitabine. In contrast, there was no association between antibiotics and survival among patients who received fluorouracil.

**Meaning:**

The findings of this study suggest that the addition of antibiotics to first-line gemcitabine chemotherapy regimens may improve outcomes for patients with metastatic PDAC.

## Teaser

This cohort study examines survival among patients receiving antibiotic therapy concurrent with chemotherapy with gemcitabine or fluorouracil for metastatic pancreatic ductal adenocarcinoma.

## Introduction

Pancreatic ductal adenocarcinoma (PDAC) is the third leading cause of cancer-related death in the US and is projected to become the second leading cause by 2030.^1,2^ The poor 5-year survival rate of 11%^1^ is due to clinical presentation at an advanced stage and limited effectiveness of standard treatment regimens. Among patients with metastatic disease, gemcitabine plus nab-paclitaxel and fluorouracil plus leucovorin, irinotecan, and oxaliplatin are the mainstays of first-line chemotherapy despite a median overall survival (OS) of less than 12 months with both treatments.^3,4^

The rapid development of multifactorial resistance to chemotherapy is an important contributor to the dismal prognosis in PDAC.^5^ Several preclinical studies have demonstrated the ability of bacteria in the tumor microenvironment to mediate chemoresistance through mechanisms including metabolization^6,7,8,9,10^ and altered immunosurveillance.^10,11^ Compared with normal pancreatic tissue, the PDAC microbiome is characterized by higher bacterial abundance and a dysbiotic bacterial profile^8,12^ enriched with Gammaproteobacteria capable of metabolizing gemcitabine into its inactive form.^8^ Some preclinical studies have similarly discovered bacteria-mediated mechanisms of fluorouracil resistance^11,13^ while others have identified bacteria-mediated mechanisms of fluorouracil activation.^7,14^ Small retrospective studies of patients with PDAC have found associations between antibiotic exposure and improved treatment outcomes, particularly among patients receiving gemcitabine.^15,16^

Collectively, these studies suggest that antibiotics may affect outcomes in patients with PDAC. We hypothesized that antibiotics with pancreatic penetration and Gammaproteobacteria coverage received in the perichemotherapy period would improve OS among patients treated with gemcitabine, but not fluorouracil. In this study we analyzed the association of antibiotic exposure in the month before or after chemotherapy initiation with survival in a large, population-based cohort of patients with metastatic PDAC treated with first-line gemcitabine or fluorouracil regimens.

## Methods

### Data Source

We analyzed data on patients with PDAC in the Surveillance, Epidemiology, and End Results (SEER)–Medicare-linked database. The SEER program provides demographic, socioeconomic, and cancer-related variables reported by 20 National Cancer Institute registries covering approximately 26% of the US population.^17^ SEER patient records are matched to Medicare inpatient (Part A), outpatient (Part B), and prescription drug (Part D) insurance claims containing information on billed diagnoses, procedures, and filled prescriptions. This study was approved by the institutional review board of the Icahn School of Medicine at Mount Sinai with a waiver of informed consent because the study used publicly available deidentified data and was considered to have minimal risk. Reporting of this study followed the guidelines set forth in the Strengthening the Reporting of Observational Studies in Epidemiology (STROBE) reporting guideline.

### Study Population

**Figure 1.**
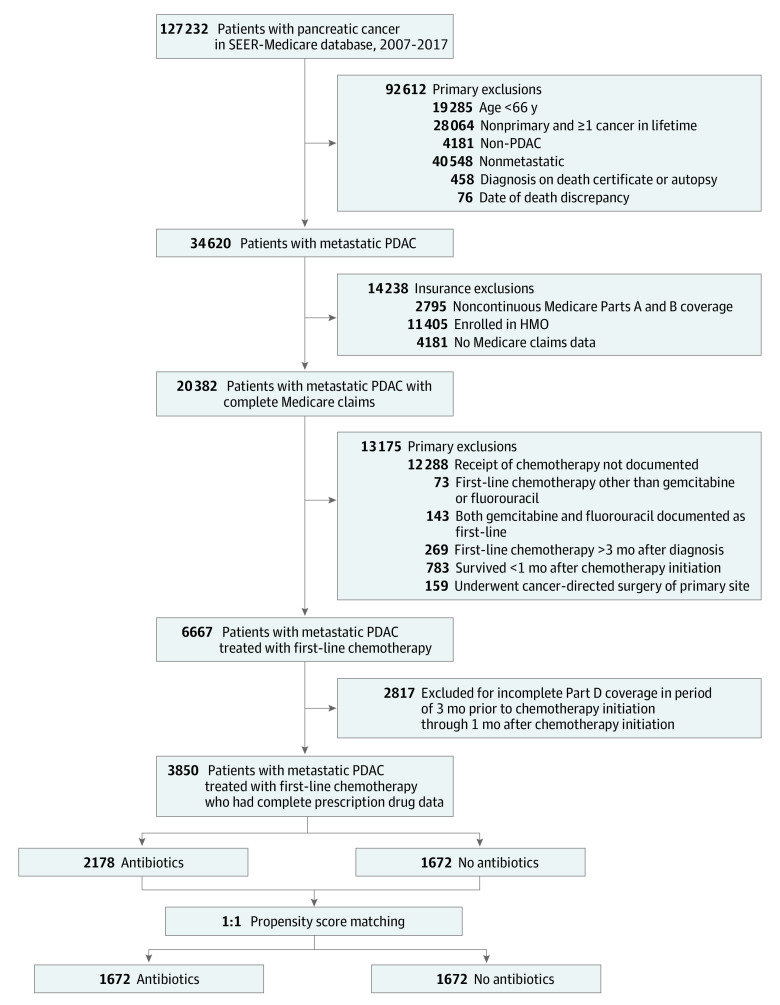
Full and Propensity Score–Matched Cohort HMO indicates health maintenance organization; PDAC, pancreatic ductal adenocarcinoma; and SEER, Surveillance, Epidemiology, and End Results.

We identified SEER-Medicare patients with a histologically confirmed diagnosis (eTable 1 in Supplement 1)^18,19,20,21^ of PDAC between January 1, 2007, and December 31, 2017, who received first-line gemcitabine- or fluorouracil-based chemotherapy (Figure 1). To ensure we captured complete medical claims between the year before diagnosis and date of death or end of study follow-up for Medicare beneficiaries who typically become eligible at age 65 years, we limited our cohort to patients aged 66 years or older with continuous Medicare Parts A and B coverage who were not enrolled in a health care maintenance organization. Similarly, Part D prescription drug plan enrollment was required in the 3 months prior to chemotherapy initiation through the month following chemotherapy initiation. We further limited our cohort to patients with unresectable metastatic disease. Patients with documented cancer-directed surgery were excluded (eTable 1 in Supplement 1). Additionally, we excluded patients with more than 1 lifetime primary cancer diagnosis and patients with cancer diagnosed at the time of death, on autopsy, or those who died before the end of the antibiotic exposure period. Exclusion criteria specific to the SEER-Medicare database are contained within the eMethods in Supplement 1.

### Treatment Characteristics

Documentation of chemotherapy and radiotherapy administration was extracted from Medicare claims data (eTable 1 in Supplement 1). To avoid misclassifying first-line treatment, patients who first received chemotherapy more than 3 months after diagnosis were excluded (Figure 1). First-line treatment was defined as the chemotherapeutic regimen received within the 30 days following the first administration of chemotherapy. Patients were classified as either having received first-line gemcitabine- or fluorouracil-based chemotherapy. To ensure accurate and clinically appropriate classification of first-line treatment, we required that patients receive either gemcitabine or fluorouracil but not both chemotherapeutic agents in the first month after initiating chemotherapy. We used an intention-to-treat approach for chemotherapy classification.

### Antibiotic Exposure

Since we hypothesized that gemcitabine-metabolizing bacteria may be associated with the rapid onset of chemoresistance,^5^ we defined antibiotic exposure as either 1 filled antibiotic prescription of 5 or more days or 1 injectable antibiotic dose between the month before and month after first receiving chemotherapy. Prior studies have used similar antibiotic day supply^16,22,23^ and exposure window definitions.^24,25,26,27^ Patients were classified as either having received or not having received antibiotics in this period. Systemic, nonophthalmic, nontopical antibiotic prescriptions and injectable antibiotic administration were identified (eTable 2 in Supplement 1).^28^ Antibiotics were further classified as having either gram-negative or no gram-negative coverage.

### Outcomes

The primary outcome of the study was OS, calculated as the time between diagnosis and death. Patients alive at the end of Medicare follow-up (December 31, 2019) were censored. We also calculated cancer-specific survival as the time between diagnosis and cancer-related death specified in SEER registry data and censored patients alive at the end of SEER follow-up (December 31, 2018) and patients whose causes of death were not attributable to cancer. Secondary outcomes included survival stratified by antibiotic coverage, class, and route of administration among gemcitabine-treated patients who received antibiotics.

### Statistical Analysis

Data analysis was conducted between September 1, 2021, and January 15, 2023. We first assessed differences in baseline sociodemographic and clinical characteristics between the antibiotic and nonantibiotic groups and calculated standardized mean differences to measure covariate balance between groups. Next, we generated propensity scores (PSs) to control for allocation bias and known confounding variables. A multivariable logistic regression model including predetermined sociodemographic and clinical covariates likely associated with antibiotic receipt and survival of patients with PDAC was used in PS estimation. Only covariates preceding antibiotic receipt were included in this model. Using Medicare claims, we identified infections in various anatomic locations and endoscopic retrograde cholangiopancreatography (eTable 3 in Supplement 1) procedures before antibiotic receipt. Additional covariates included sex, age at diagnosis, US geographic census region, residential population density,^29^ Yost US-based socioeconomic status quintiles,^30,31^ tumor location, radiotherapy, time from diagnosis to chemotherapy initiation, and the Charlson Comorbidity Index,^32^ which was calculated to adjust for differences in underlying health problems within the cohort. Data on race and ethnicity were also obtained to detect differences in proportions that may affect the generalizability of the findings. Multiple imputation by chained equations was used to predict missing values for race and ethnicity (0.1%), Charlson Comorbidity Index (0.8%), and Yost quintiles (4%).

To further reduce confounding and simulate patient randomization, we performed PS pair-matching of patients receiving antibiotic therapy with those not receiving antibiotics using optimal matching techniques that maximize PS balance and cohort size (eFigure 1 in Supplement 1).^33^ We generated Kaplan-Meier curves to compare survival stratified by antibiotic status in the unmatched and PS-matched cohorts. To estimate the association between antibiotic receipt and OS, we fit Cox proportional hazards regression models with robust variance estimators to account for clustering in the PS-matched cohort. Time-independent Schoenfeld residuals confirmed assumptions of proportionality (eTable 6 in Supplement 1). Additionally, we fit inverse probability of treatment-weighted (IPTW) Cox proportional hazards regression models in the unmatched cohort to account for allocation bias in the use of antibiotics while avoiding the sample size reduction associated with PS matching. Weights were calculated from PSs, and patients with weights greater than the 99th percentile were excluded.^34^

We analyzed cancer-specific survival using Fine-Gray competing risk models in the matched cohort and cause-specific regression models for IPTW analyses in the unmatched cohort. We fit additional Cox proportional hazards regression models to analyze associations of individual antibiotic attributes with OS among gemcitabine-treated patients who received antibiotics. All hypothesis tests were 2-sided, and the a priori statistical significance threshold was set at *P* < .05. Statistical analyses were conducted using SAS, version 9.4 (SAS Institute Inc) and R, version 4.1.0 (R Foundation for Statistical Computing) statistical packages.

## Results

We identified 3850 patients with unresectable, metastatic PDAC treated with first-line gemcitabine or fluorouracil therapy between January 1, 2007, and December 31, 2017 (Figure 1). Perichemotherapy antibiotic receipt was identified in 2178 (56.6%) patients (Table 1). The mean (SD) age at diagnosis was 74.2 (5.8) years. Patients included 2102 women (54.6%) and 1748 men (45.4%). Most were White (3396 [88.2%]), from metropolitan areas (3393 [88.1%]) in the northeastern or western US (2952 [76.7%]), and disproportionately belonged to the highest socioeconomic quintile (1407 [36.5%]). Overall, most patients received first-line gemcitabine-based chemotherapy (3150 [81.8%]); few received fluorouracil (700 [18.2%]). Before 2012, patients predominantly received gemcitabine (1229 [92.0%]), with an increase in the proportion of patients treated with fluorouracil occurring after 2011 (593 [23.6%]) (eTable 4 in Supplement 1). Antibiotic receipt was more common in patients with prior infections (879 [40.4%] vs 323 [19.3%]) who underwent endoscopic retrograde cholangiopancreatography (537 [29.2%] vs 284 [17.0%]) and whose tumors were in the pancreatic head (849 [39.0%] vs 549 [32.8%]). Characteristics of the gemcitabine-treated and fluorouracil-treated subgroups are presented in eTable 4 in Supplement 1. The balance of baseline sociodemographic and clinical variables between study groups improved with PS-matching (eTable 5 in Supplement 1).

**Table 1.** Baseline Demographic and Clinical Characteristics of Patients With Metastatic PDAC

Characteristic	No. (%)		SMD
	Antibiotics (n = 2178)	No antibiotics (n = 1672)	
Age at diagnosis, mean (SD), y	73.9 (5.7)	74.6 (5.9)	.13
Sex			
Female	1188 (54.5)	914 (54.7)	.002
Male	990 (45.5)	758 (45.3)	
Race^a^			
Black	131 (6.0)	129 (7.7)	.01
White	1951 (89.6)	1445 (86.4)	
Other	96 (4.4)	98 (5.9)	
Census region			.18
West	722 (33.1)	487 (29.1)	
Midwest	202 (9.3)	129 (7.7)	
Northeast	903 (41.5)	840 (50.2)	
South	351 (16.1)	216 (12.9)	
Population density			
Metropolitan area	1907 (87.6)	1486 (88.9)	.04
Nonmetropolitan area	271 (12.4)	186 (11.1)	
Yost SES index, %			
0-20	231 (10.6)	205 (12.3)	.08
>20-40	320 (14.7)	206 (12.3)	
>40-60	371 (17.0)	282 (16.9)	
>60-80	459 (21.1)	369 (22.1)	
>80-100	797 (36.6)	610 (36.5)	
Year of diagnosis			
2007-2009	394 (18.1)	391 (23.4)	.17
2010-2012	491 (22.5)	383 (22.9)	
2013-2015	704 (32.3)	548 (32.8)	
2016-2017	589 (27.0)	350 (20.9)	
Site of disease			
Head	849 (39.0)	549 (32.8)	.14
Body and neck	451 (20.7)	384 (23.0)	
Tail	376 (17.3)	350 (20.9)	
Unspecified	502 (23.0)	389 (23.3)	
Radiotherapy			
Yes	88 (4.0)	80 (4.8)	.04
No	2090 (96.0)	1592 (95.2)	
Time to chemotherapy, mean (SD), wk	4.5 (2.8)	4.5 (2.9)	.005
Charlson Comorbidity Index			
0	809 (37.1)	632 (37.8)	.02
1	656 (30.1)	502 (30.0)	
>1	713 (32.7)	538 (32.2)	
ERCP			
Yes	637 (29.2)	284 (17.0)	.29
No	1541 (70.8)	1388 (83.0)	
Infection			
Any location	879 (40.4)	323 (19.3)	.47
Intraabdominal	206 (9.5)	74 (4.4)	.20
Respiratory	221 (10.1)	88 (5.3)	.18
Genitourinary	391 (18.0)	133 (8.0)	.30
Blood	226 (10.4)	78 (4.7)	.22
Skin	175 (8.0)	35 (2.1)	.27
Other bacterial	156 (7.2)	54 (3.2)	.18
First-line chemotherapy^b^			
Gemcitabine	1741 (79.9)	1409 (84.3)	.11
Fluorouracil	437 (20.1)	263 (15.7)	

Abbreviations: ERCP, endoscopic retrograde cholangiopancreatography; PDAC, pancreatic ductal adenocarcinoma; SES, socioeconomic status; SMD, standardized mean difference.

^a^ Variable with the least amount of missing data was used to categorize race and ethnicity as Black, White, and other (American Indian/Alaska Native, Asian/Pacific Islander).

^b^ Chemotherapy type was not included in propensity score estimation.

**Figure 2.**
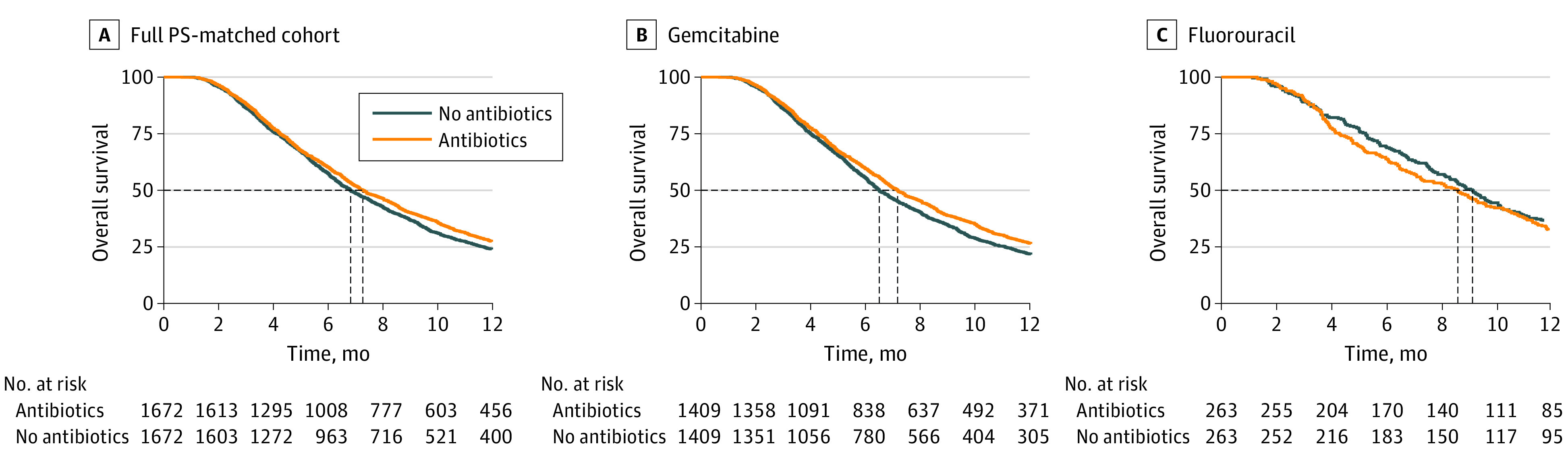
Association of Antibiotic Receipt With Survival in Propensity Score (PS)–Matched Cohort Twelve-month survival for patients who received and did not receive antibiotics in the 2-month perichemotherapy exposure period. A, In the full PS-matched cohort, median survival with antibiotics, 7.3 (95% CI, 6.9-7.7) months; no antibiotics, 6.8 (95% CI, 6.5-7.2) months; log rank *P* = .03. B, In the gemcitabine cohort, median survival with antibiotics, 7.1 (95% CI, 6.8-7.5) months; no antibiotics, 6.5 (95% CI, 6.2-6.8) months; log rank *P* = .003. C, in the fluorouracil cohort, median survival with antibiotics, 8.6 (95% CI, 7.3-9.7) months; no antibiotics, 9.2 (95% CI, 8.3-10.1) months; log rank *P* = .41. Dashed lines show median survival times for each group. Survival was calculated as months since diagnosis.

In the cohort of 1672 PS-matched patient pairs (76.8% of the antibiotic group), median survival among all patients who received peritreatment antibiotics was 7.3 (95% CI, 6.9-7.7) months compared with 6.8 (95% CI, 6.5-7.2) months among patients who did not receive antibiotics (Figure 2). When stratified by first-line chemotherapy, median survival for gemcitabine-treated patients in the antibiotic group was 7.1 (95% CI, 6.8-7.5) months compared with 6.5 (95% CI, 6.2-6.8) months for patients in the nonantibiotic group. Among fluorouracil-treated patients, median survival for the antibiotic group was 8.6 (95% CI, 7.3-9.7) months compared with 9.2 (95% CI, 8.3-10.1) months for the nonantibiotic group. All but 34 patients died by the end of study follow-up. Analyses of median survival before PS-matching revealed similar results (eFigure 2 in Supplement 1).

In PS-matched Cox proportional hazards regression analyses, receipt of peritreatment antibiotics was associated with improvement in OS (Table 2) (hazard ratio [HR], 0.93; 95% CI, 0.86-0.99; *P* = .03). On stratification by chemotherapy, we found antibiotic receipt among gemcitabine-treated patients to be associated with a greater improvement in OS (HR, 0.89; 95% CI, 0.83-0.96; *P* = .003). However, antibiotic receipt was not associated with a significant difference in OS among patients treated with first-line fluorouracil (HR, 1.08; 95% CI, 0.90-1.29; *P* = .41). Consistent with analyses of OS, antibiotic receipt among gemcitabine-treated patients was associated with improved cancer-specific survival (HR, 0.84; 95% CI, 0.77-0.92; *P* < .001). Among patients treated with fluorouracil, antibiotic status was not associated with a difference in cancer-specific survival (HR, 1.12; 95% CI, 0.90-1.36; *P* = .29). To account for potential biases introduced by incomplete matching, we performed IPTW analyses in the unmatched cohort of 3811 patients after excluding patients with weights greater than the 99th percentile, which revealed similar results in analyses of OS and cancer-specific survival (Table 2).

**Table 2.** Survival Outcomes Associated With Antibiotic Receipt

Antibiotic group outcome	PS-matched HR (95% CI)	*P* value	IPTW HR (95% CI)	*P* value
**Overall survival**				
All chemotherapy	0.93 (0.86-0.99)	.03	0.91 (0.85-0.97)	.005
Gemcitabine	0.89 (0.83-0.96)	.003	0.90 (0.84-0.97)	.006
Fluorouracil	1.08 (0.90-1.29)	.41	0.98 (0.83-1.14)	.76
**Cancer-specific survival**				
All chemotherapy	0.89 (0.82-0.96)	.004	0.85 (0.78-0.92)	<.001
Gemcitabine	0.84 (0.77-0.92)	<.001	0.83 (0.76-0.91)	<.001
Fluorouracil	1.12 (0.90-1.36)	.29	0.97 (0.79-1.18)	.74

Abbreviations: HR, hazard ratio; IPTW, inverse probability of treatment weighting; PS, propensity score.

We further sought to understand whether antibiotic coverage, class, or route of administration could explain the improved survival rate among gemcitabine-treated patients who received antibiotics. Within this subgroup of 1741 patients, 93.3% received antibiotics with gram-negative coverage, which was not associated with differential survival outcomes compared with patients who received antibiotics without gram-negative coverage (Table 3) (HR, 1.00; 95% CI, 0.83-1.21; *P* > .99). Most antibiotics were nonpenicillin β-lactams (50.5%) or fluoroquinolones (42.2%), whereas a larger proportion of fluorouracil-treated patients received nonpenicillin β-lactams (71.2%). Nonpenicillin β-lactams were associated with an 11% reduction in risk of death (Table 3) (HR, 0.89; 95% CI, 0.81-0.97; *P* = .01) compared with all other antibiotic classes, while fluoroquinolones alone were not associated with a difference in OS (HR, 1.00; 95% CI, 0.93-1.12; *P* = .70). Next, we evaluated route of administration (Table 3). Compared with patients who only received oral antibiotics, patients who received injectable antibiotics had an HR of 0.91 (95% CI, 0.82-1.00; *P* = .05). Of all gemcitabine-treated patients who received injectable antibiotics, 76.9% received nonpenicillin β-lactams.

**Table 3.** Association of Antibiotic Properties With Overall Survival Among Patients Receiving Gemcitabine^a^

Antibiotic property	Sample size, No. (% of total)	Overall survival, HR (95% CI)	*P* value
Coverage class			
Gram-negative	1624 (93.3)	1.00 (0.83-1.21)	>.99
Nonpenicillin β-lactams^b^	880 (50.5)	0.89 (0.81-0.97)	.01
Quinolones	734 (42.2)	1.02 (0.93-1.12)	.70
Penicillins	287 (16.5)	1.04 (0.91-1.18)	.59
Macrolides	155 (8.9)	1.06 (0.90-1.26)	.46
Tetracyclines	98 (5.6)	1.17 (0.96-1.44)	.13
Sulfonamides	91 (5.2)	1.09 (0.89-1.35)	.42
Aminoglycosides	31 (1.8)	1.00 (0.70-1.43)	>.99
Other antibiotics^c^	247 (14.2)	1.15 (1.00-1.31)	.05
Route			
Injectable vs oral	966 (55.5)	0.91 (0.82-1.00)	.05

Abbreviation: HR, hazard ratio.

^a^ Overall survival stratified by antibiotic properties among patients treated with first-line gemcitabine-based chemotherapy who received antibiotics (n = 1741). Antibiotic attributes do not sum to the total because some patients received more than 1 antibiotic.

^b^ Includes cephalosporin, carbapenem, and monobactam antibiotics.

^c^ Includes lincosamide, glycopeptide, lipoglycopeptide, oxazolidinone, streptogramin, rifamycin, and nitroimidazole antibiotics.

To evaluate the validity of our findings, we performed several sensitivity analyses. We found that altering minimal oral prescription duration definitions of antibiotic receipt did not change the association with OS (eTable 7 in Supplement 1). In a sensitivity analysis within the cohort defining antibiotic exposure as receipt of antibiotics between diagnosis and death, we observed that more than 20% of the patients first received antibiotics at a date beyond the median OS, thereby introducing survivorship bias (HR, 0.67; 95% CI, 0.62-0.73). Additionally, we observed that including all patients with sepsis diagnoses in the antibiotic group, irrespective of documented antibiotic receipt, did not significantly change OS (eTable 8 in Supplement 1).

## Discussion

In this large retrospective cohort study of SEER-Medicare patients with metastatic PDAC, we found that receipt of antibiotics between the month before and after beginning first-line chemotherapy was associated with improved OS and cancer-specific survival among patients who received gemcitabine, but not fluorouracil. A prior investigation of human PDAC microbiome composition found that the majority (52%) of intratumoral bacterial species belong to the Gammaproteobacteria class, which produce a long isoform of cytidine deaminase (CDD_L_) capable of deaminating gemcitabine (2′,2′-difluorodeoxycytidine) into its inactive form (2′,2′-difluorodeoxyuridine).^8^ In a mouse model colonized with CDD_L_-expressing *Escherichia coli*, cotreatment with ciprofloxacin and gemcitabine induced a significant antitumor response while gemcitabine alone resulted in rapid tumor progression.^8^ Furthermore, in a multicenter retrospective study of 211 patients with PDAC undergoing pancreatoduodenectomy, adjuvant gemcitabine therapy was associated with an improvement in progression-free survival only among patients whose bile was not colonized with *Klebsiella pneumoniae*, a subspecies of Gammoproteobacteria.^15^ Preclinical studies have also identified bacteria-mediated molecular mechanisms of fluorouracil chemoresistance through autophagy activation^11^ and fluorouracil metabolization by bacterial thymidine phosphorylases.^13^ However, other studies have noted the ability of bacteria to activate fluorouracil prodrugs^7^ and enhance fluorouracil efficacy through ribonucleotide metabolism,^14^ suggesting that disruption of bacterial composition may substantially alter the response to fluorouracil.

In what is, to our knowledge, the only study to date to explore the association between antibiotic receipt and survival of patients with metastatic PDAC, a single-center, retrospective cohort analysis of 118 gemcitabine-treated and 98 fluorouracil-treated patients revealed that receipt of 7 or more days of antibiotics between diagnosis and death was associated with improved OS for patients treated with gemcitabine (HR, 0.40), but not fluorouracil (HR, 1.17).^16^ In a sensitivity analysis within the cohort, use of a similar antibiotic exposure period between diagnosis and death introduced survivorship bias and resulted in an overestimation of the role of antibiotic receipt in survival. Therefore, we selected a specific antibiotic exposure period to avoid compromising the internal validity of our study and evaluate the importance of antibiotic administration timing relative to chemotherapy administration, which had yet to be explored. Given the rapid onset of chemoresistance observed clinically,^5^ we hypothesized that antibiotic receipt between the month before and after chemotherapy initiation would maximally impact OS. Prior studies that investigated the association between antibiotics and response to immune checkpoint inhibition therapies in patients with PDAC used similar peritreatment exposure periods.^24,25,26,27^ We observed a more pronounced association between antibiotic use and OS over time (Figure 2), suggesting that antibiotics may mediate durable improvements in survival among patients treated with first-line gemcitabine.

Our findings of an association between antibiotic receipt and improved survival specific to patients treated with gemcitabine are consistent with the results of prior studies and provide further support for the clinical relevance of bacteria-mediated gemcitabine resistance.^8,9^ Antibiotic-mediated elimination of CDD_L_-expressing Gammaproteobacteria in the PDAC microenvironment may underlie the observed improvement in survival among gemcitabine-treated patients. Were an independent antibiotic mechanism responsible for the observed improvement in OS, we would have expected a similar survival benefit among patients treated with fluorouracil.

We further explored whether specific antibiotic properties were associated with the improvement in survival among gemcitabine-treated patients who received antibiotics. We hypothesized that antibiotics with coverage against gram-negative Gammaproteobacteria might be associated with a greater survival benefit. However, our study was underpowered to detect coverage-specific differences in OS, because 93.3% of subgroup patients received antibiotics with some gram-negative coverage (Table 3). On stratification by antibiotic class, we found that nonpenicillin β-lactams were associated with improved survival compared to all other antibiotics classes (Table 3), which may be due to their excellent pancreatic penetration and gram-negative coverage.^35^ We also observed a borderline, nonsignificant improvement in OS among patients who received any injectable antibiotics compared with only oral antibiotics, potentially reflecting route-dependent differences in pancreatic penetration or the widespread receipt of injectable nonpenicillin β-lactams in this cohort.

### Strengths and Limitations

We carefully used methods to minimize biases and threats to validity in this large, population-based, retrospective cohort study. Propensity score estimation was used to address allocation bias and pair-matching further reduced imbalances in confounding baseline characteristics between the antibiotic and nonantibiotic groups. The consistently observed improvement in survival among gemcitabine-treated patients who received antibiotics across PS-matched and IPTW analyses signifies the robustness of these findings. Furthermore, given that antibiotic receipt often reflects a poor state of health, we believe that the reported survival benefit associated with peritreatment antibiotics in this study may underestimate the potential benefit of coadministering gemcitabine and antibiotics.

Despite its many strengths, this study had several limitations. Patients in this study were predominantly older, White, from the northeastern or western US, and disproportionately belonged to the highest socioeconomic quintile. Additionally, there were potential unmeasured confounders, including patient contraindications to antibiotic use and differences in intratumoral microbial composition that may have influenced these results. We were further limited by the inability to directly link antibiotic prescriptions to medical claims using Medicare data. Therefore, it was not possible to conclude reasons for antibiotic receipt that may have directly influenced survival outcomes, and further, use of antibiotics was unlikely to be random. However, we included infection diagnoses before antibiotic receipt in PS estimation to address potential differences in acute health status between patients. We observed that many patients with documented bacterial infections did not receive 5 or more days of antibiotics. This may have been due to our 5-day or more supply definition of antibiotic receipt, incomplete antibiotic capture, or inaccuracies in medical claims coding. In a sensitivity analysis, no significant changes were observed on lowering the antibiotic day supply requirement to 3 or more days (eTable 7 in Supplement 1). To estimate how incomplete antibiotic capture might have impacted our findings, we conducted a sensitivity analysis including all patients with sepsis claims in the antibiotic group irrespective of documented antibiotic administration. We did not observe significant changes in OS (eTable 8 in Supplement 1), further suggesting the robustness of these results. In addition, the association between antibiotic receipt and survival was relatively small. However, it was consistent across analyses and statistically significant in most instances.

## Conclusions

Herein, we report the results of a large retrospective cohort study using national data from the SEER-Medicare–linked database to better understand the association of antibiotics with PDAC survival. We found that perichemotherapy antibiotics were associated with improved survival among patients treated with first-line gemcitabine, but not fluorouracil, suggesting a potential role for perichemotherapy antibiotic treatment in patients with metastatic PDAC receiving gemcitabine. We recommend that prospective studies investigate the effect of perichemotherapy administration of antibiotics with high pancreatic penetration and gram-negative coverage on survival in diverse populations of patients with metastatic PDAC treated with gemcitabine.

## Notes

Supplement 1. **eMethods.** Exclusion Criteria **eTable 1.** Codes Used in Cohort Creation and Treatment Identification **eTable 2.** Antibiotic Codes **eTable 3.** ERCP and Infection Codes **eTable 4**. Characteristics of the Gemcitabine and Fluorouracil Subgroups **eTable 5.** Baseline Demographic and Clinical Characteristics of PS-Matched Cohort **eTable 6.** Result of Schoenfeld Test in Propensity-Matched Analyses **eTable 7.** Sensitivity Analysis Using Varying Antibiotic Receipt Definitions **eTable 8.** Sensitivity Analysis for Infection Capture **eFigure 1.** Propensity Score Distribution Before and After Matching **eFigure 2.** Kaplan-Meier Curves in the Unmatched Cohort
eReferences

Supplement 2.
Data Sharing Statement
