# Supplementary material for: Association of Antibiotic Receipt With Survival Among Patients With Metastatic Pancreatic Ductal Adenocarcinoma Receiving Chemotherapy
Source: JAMA Netw Open. 2023 Mar 23;6(3):e234254. doi: 10.1001/jamanetworkopen.2023.4254 (PMC10037151; doi:10.1001/jamanetworkopen.2023.4254)
Supplement: Supplement 1. — eMethods. Exclusion Criteria eTable 1. Codes Used in Cohort Creation and Treatment Identification eTable 2. Antibiotic Codes eTable 3. ERCP and Infection Codes eTable 4. Characteristics of the Gemcitabine and Fluorouracil Subgroups eTable 5. Baseline Demographic and Clinical Characteristics of PS-Matched Cohort eTable 6. Result of Schoenfeld Test in Propensity-Matched Analyses eTable 7. Sensitivity Analysis Using Varying Antibiotic Receipt Definitions eTable 8. Sensitivity Analysis for Infection Capture eFigure 1. Propensity Score Distribution Before and After Matching eFigure 2. Kaplan-Meier Curves in the Unmatched Cohort eReferences [file jamanetwopen-e234254-s001.pdf]

## Supplementary Online Content

Fulop DJ, Zylberberg HM, Wu YL, et al. Association of antibiotic receipt with survival among patients with metastatic pancreatic ductal adenocarcinoma receiving chemotherapy. *JAMA Netw Open*. 2023;6(3):e234254. doi:10.1001/jamanetworkopen.2023.4254

**eMethods.** Exclusion Criteria

**eTable 1.** Codes Used in Cohort Creation and Treatment Identification

**eTable 2.** Antibiotic Codes

**eTable 3.** ERCP and Infection Codes

**eTable 4**. Characteristics of the Gemcitabine and Fluorouracil Subgroups

**eTable 5.** Baseline Demographic and Clinical Characteristics of PS-Matched Cohort

**eTable 6.** Result of Schoenfeld Test in Propensity-Matched Analyses

**eTable 7.** Sensitivity Analysis Using Varying Antibiotic Receipt Definitions

**eTable 8.** Sensitivity Analysis for Infection Capture

**eFigure 1.** Propensity Score Distribution Before and After Matching

**eFigure 2.** Kaplan-Meier Curves in the Unmatched Cohort

**eReferences**

This supplementary material has been provided by the authors to give readers additional information about their work.

© 2023 Fulop DJ et al. *JAMA Network Open.*

**eMethods. Exclusion Criteria**

We excluded patients with a documented date of death in SEER but not Medicare because of discrepant vital status at the end of study follow-up (December 31, 2019; Figure 1). Since SEER-Medicare patients on average have >150 total Medicare claims in the year before and after cancer diagnosis,[1] patients without a single billed Medicare claim were considered to have incomplete data and excluded from the study cohort.

© 2023 Fulop DJ et al. *JAMA Network Open.*

**eTable 1.** Codes used in cohort creation and treatment identification

| Variable | | Codes |
|---|---|---|
| Histology | | ICD-O-3: 8000, 8010, 8020-8022, 8050, 8140, 8141, 8143, 8147, 8211, 8230, 8260, 8440, 8441, 8450, 8453, 8470, 8471, 8480, 8481, 8500, 8503, 8521, 8550, 8560 |
| Chemotherapy | General Administration | ICD-9-CM: V58.1, 99.25<br>ICD-10-CM: Z51.11<br>ICD-10-PCS: 3E03305, 3E04305<br>HCPCS: 96400-96549, G0498, Q0083-Q0085<br>Revenue Center: 0331, 0332, 0335 |
| | Gemcitabine | HCPCS: J9198, J9199, J201 |
| | Fluorouracil | HCPCS: J9190<br>NDC: 63323011710, 63323011751, 63323011761 |
| | Irinotecan | HCPCS: J9205, J9206, C9474 |
| | Oxaliplatin | HCPCS: J9263 |
| | Paclitaxel | HCPCS: J9264, J9265, J9267 |
| | Capecitabine | HCPCS: J8520, J8521<br>NDC: 72205000792, 54868526002, 59651020508, 00004110020, 00004110075, 00004110150, 00004110151, 00004110175, 00054027121, 00054027223, 00093747306, 00093747489, 00378251191, 00378251278, 16714046701, 16714046801, 42291019060, 42291019112, 54868414300, 64980027712, 65162084306, 65162084416, 16729007329, 16729007212 |
| Surgery | | ICD-9-CM: 52.51, 52.6, 52.7<br>ICD-10-PCS: 0FBG0ZZ, 0DT90ZZ, 0FTG0ZZ<br>HCPCS: 48150, 48152, 48153, 48154, 48155 |
| Radiation therapy | | ICD-9-CM: V58.0, V66.1, V67.1, 92.21-92.29<br>ICD-10-PCS: Z51.0, Z51.89, Z08<br>HCPCS: 77014, 77371-77373, 77385-77387, 77401-77499, 77520-77525, 77750-77799, G0173, G0251, G0256, G0261, G0339, G0340, G6001-G6017, 0082T<br>Revenue Center: 0330, 0333 |

To determine each patient's first course of chemotherapy ICD, HCPCS, and NDC codes listed in the Cancer Medications Enquiry Database (CanMED)[2] were extracted from Medicare inpatient, outpatient, and physician files. Abbreviations: ICD-O-3, International Classification of Diseases for Oncology, Third Edition; HCPCS, Level II Healthcare Common Procedure Coding System; NDC, National Drug Code; ICD-9, International Classification of Disease, Ninth Revision; ICD-10, International Classification of Disease, Tenth Revision, Clinical Modification; CM, Clinical Modification; PCS, Procedure Coding System.

© 2023 Fulop DJ et al. *JAMA Network Open.*

**eTable 2.** Antibiotic codes

| Antibiotic Class | ATC-4 Class | HCPCS Codes |
|---|---|---|
| Aminoglycosides | J01G | J0278, J0291, J1580, J1840, J1850, J3000, J3260, J3320 |
| Amphenicols | J01B | J0720 |
| Non-Penicillin Beta-Lactams (Cephalosporins, Carbapenems, Monobactams) | J01D | J0690, J0692-J0699, J0710, J0712-J0715, J0742, J0743, J1267, J1335, J1890, J2185, J2186 |
| Macrolides, Lincosamides, and Streptogramins | J01F | J0456, J1364, J2010 |
| Penicillins | J01C | J0290, J0295, J0558, J0561, J2510, J2540, J2543, J2700 |
| Quinolones | J01M | J0744, J1590, J1956, J2280 |
| Sulfonamides and Trimethoprim | J01E | |
| Tetracyclines | J01A | J0120-J0122, J2265, J2460, J3243 |
| Other Antibiotics (Lincosamides, Glycopeptides, Lipoglycopeptides, Oxazolidinones, Streptogramins, Rifamycins, Nitroimidazoles) | J01X | J0770, J0875, J0878, J2020, J2406, J2407, J3090, J3095, J3370 |

All unique NDC codes present in the Medicare Part D Events files were mapped to Anatomical Therapeutic Chemical Level 4 (ATC-4) classes using an algorithm[3] that queries the National Library of Medicine RxNorm application programming interface (API). We confirmed antibiotic class mappings to be 100 percent accurate using generic or brand antibiotic drug names where populated. ATC-4 codes corresponding to major antibiotic classes were identified. HCPCS codes were used to identify receipt of injectable antibiotics. Abbreviations: Anatomical Therapeutic Chemical Class Level 4; HCPCS, Level II Healthcare Common Procedure Coding System.

**eTable 3.** ERCP and infection codes

| Variable | | Codes |
|---|---|---|
| ERCP | | ICD-9-CM: 51.10, 51.11, 51.84-51.87, 51.99, 52.13, 52.93<br>ICD-10-PCS: BF110ZZ, BF111ZZ, BF11YZZ, BF100ZZ, BF101ZZ, BF10YZZ, 0F998ZZ, 0F9C8ZZ, 0F9C80Z, 0FHB8DZ, 0F798DZ, 0FR98JZ, 0FHD8DZ<br>HCPCS: 74328-74330, 43260-43269, 43271, 43272 |
| Infections | Intra-abdominal | ICD-9-CM: 008.45, 540.0-540.9, 541, 542, 567.0, 567.1, 567.21-567.29, 567.31, 567.38, 567.81, 567.89, 567.9, 569.5, 576.1, 575.0, 575.10-575.12, 590.2<br>ICD-10-CM: A04.72, K35.2, K35.3, K35.80, K35.89, K57.0, K57.21, K57.41, K63.0, K65.0-K65.9, K68.12, K68.19, K81.0-K81.2, K81.9, K80.30-K80.37, K83.0, K83.09, N15.1 |
| | Respiratory | ICD-9-CM: 466.0, 480, 481, 482.00-482.49, 482.80-482.89, 483.1-483.8, 484.1-484.8, 485, 486, 487.0, 513.0<br>ICD-10-CM: A22.1 A37.91, A48.1, B44.0, J11.00, J13, J14, J15.0-J15.9, J16.0, J16.8, J17, J18.0-J18.9, J20.9, J85.1<br>DRG: 193-195 |
| | Genitourinary | ICD-9-CM: 590.1, 590.10, 590.11, 590.3, 590.9, 595.0, 599.0, 996.64<br>ICD-10-CM: N10, N15.9, N28.84-N28.86, N30.00, N30.01, N39.0, T83.51XA |
| | Blood | ICD-9-CM: 038.0, 038.10-038.12, 038.19, 038.2, 038.3, 038.40-038.49, 038.8, 038.9, 995.91, 995.92<br>ICD-10-CM: A40.3, A40.9, A41.01, A41.02, A41.1, A41.2, A41.3, A41.4, A41.5, A41.89, A41.9, R65.20, R65.21 |
| | Skin | ICD-9-CM: 681, 681.00, 681.110, 681.9, 682.0-682.9<br>ICD-10-CM: K12.2, L03.019, L03.039, L03.119, L03.211, L03.221, L03.317, L03.319, L03.811, L03.818, L03.90 |
| | Other Bacterial Etiology | ICD-9-CM: 041.0-041.8<br>ICD-10-CM: A48.0-A48.99 A49.01, A49.02, A49.3, B95.00-B96.89 |

Abbreviations: ERCP, Endoscopic Retrograde Cholangiopancreatography; ICD-9, International Classification of Disease, Ninth Revision; ICD-10, International Classification of Disease, Tenth Revision, Clinical Modification; CM, Clinical Modification; PCS, Procedure Coding System; HCPCS, Level II Healthcare Common Procedure Coding System; NDC, National Drug Code; DRG, Diagnosis Related Group.

© 2023 Fulop DJ et al. *JAMA Network Open.*

**eTable 4.** Characteristics of the gemcitabine and Fluorouracil subgroups

| **Characteristics** *No. (%)* | | **Gemcitabine** (*n* = 3,150) | **Fluoruracil** (*n* = 700) |
|---|---|---|---|
| Age at Diagnosis (mean (SD)) | Years | 74.8 (5.9) | 71.8 (4.9) |
| Gender | Female | 1,755 (55.7) | 347 (49.6) |
| | Male | 1,395 (44.3) | 353 (50.4) |
| Race | White | 2764 (87.7) | 632 (90.3) |
| | Black | 224 (7.1) | 36 (5.1) |
| | Other | 162 (5.1) | 32 (4.6) |
| Census Region | West | 1,023 (32.5) | 186 (26.6) |
| | Midwest | 270 (8.6) | 61 (8.7) |
| | Northeast | 1,386 (44.0) | 357 (51.0) |
| | South | 471 (15.0) | 96 (13.7) |
| Population Density | Metro Area | 2,765 (87.8) | 628 (89.7) |
| | Non-Metro Area | 385 (12.2) | 72 (10.3) |
| Yost SES Index | 0-20% | 358 (11.4) | 78 (11.1) |
| | 20-40% | 450 (14.3) | 76 (10.9) |
| | 40-60% | 565 (17.9) | 88 (12.6) |
| | 60-80% | 691 (21.9) | 137 (19.6) |
| | 80-100% | 1,086 (34.5) | 321 (45.9) |
| Year of Diagnosis | 2007-2009 | 749 (23.8) | 36 (5.1) |
| | 2010-2012 | 718 (22.8) | 156 (22.3) |
| | 2013-2015 | 970 (30.8) | 282 (40.3) |
| | 2016-2017 | 713 (22.6) | 226 (32.3) |
| Site of Disease | Head | 1,141 (36.2) | 257 (36.7) |
| | Body & Neck | 672 (21.3) | 163 (23.3) |
| | Tail | 586 (18.6) | 140 (20.0) |
| | Unspecified | 751 (23.8) | 140 (20.0) |
| Radiation | Yes | 103 (3.3) | 65 (9.3) |
| | No | 3,047 (96.7) | 635 (90.7) |
| Time to Chemotherapy (mean (SD)) | Weeks | 4.5 (2.9) | 4.5 (2.8) |
| Charlson Comorbidity Index | 0 | 1,129 (35.8) | 312 (44.6) |
| | 1 | 941 (29.9) | 217 (31.0) |
| | >1 | 1,080 (34.3) | 171 (24.4) |
| ERCP | Yes | 765 (24.3) | 156 (22.3) |
| | No | 2,385 (75.7) | 544 (77.7) |
| Infection | Any Location | 1,013 (32.2) | 189 (27.0) |
| | Intra-Abdominal | 235 (7.5) | 45 (6.4) |
| | Respiratory | 265 (8.4) | 44 (6.3) |
| | Genitourinary | 433 (13.7) | 133 (8) |
| | Blood | 260 (8.3) | 44 (56.3 |
| | Skin | 192 (6.1) | 18 (2.6) |
| | Other Bacterial | 176 (5.6) | 34 (4.9) |

© 2023 Fulop DJ et al. *JAMA Network Open.*

Baseline demographic and clinical characteristics of the patient subgroups treated with gemcitabine or fluorouracil. Summary statistics are represented as the *n* (%) for categorical variables and mean (SD) for continuous variables. Abbreviations: SD, standard deviation; ERCP, Endoscopic Retrograde Cholangiopancreatography; SES, socioeconomic status.

© 2023 Fulop DJ et al. *JAMA Network Open.*

**eTable 5.** Baseline demographic and clinical characteristics of PS-matched cohort

| **Characteristics** *No. (%)* | | **Antibiotics (*n* = 1,672)** | **No Antibiotics (*n* = 1,672)** | **SMD** |
|---|---|---|---|---|
| Age at Diagnosis (mean (SD)) | Years | 74.2 (5.7) | 74.6 (5.9) | 0.08 |
| Gender | Female | 904 (54.1) | 914 (54.7) | 0.01 |
| | Male | 768 (45.9) | 758 (45.3) | |
| Race | White | 1,483 (88.7) | 1,445 (86.4) | 0.07 |
| | Black | 108 (6).5 | 129 (7.7) | |
| | Other | 81 (4.8) | 98 (5.9) | |
| Census Region | West | 543 (32.5) | 487 (29.1) | 0.13 |
| | Midwest | 136 (8.1) | 129 (7.7) | |
| | Northeast | 733 (43.8) | 840 (50.2) | |
| | South | 260 (15.6) | 216 (12.9) | |
| Population Density | Metro Area | 1,472 (88.0) | 1,486 (88.9) | 0.03 |
| | Non-Metro Area | 200 (12.0) | 186 (11.1) | |
| Yost SES Index | 0-20% | 184 (11.0) | 205 (12.3) | 0.06 |
| | 20-40% | 230 (13.8) | 206 (12.3) | |
| | 40-60% | 299 (17.9) | 282 (16.9) | |
| | 60-80% | 350 (20.9) | 369 (22.1) | |
| | 80-100% | 609 (36.4) | 610 (36.5) | |
| Year of Diagnosis | 2007-2009 | 330 (19.7) | 391 (23.4) | 0.10 |
| | 2010-2012 | 390 (23.3) | 383 (22.9) | |
| | 2013-2015 | 552 (33.0) | 548 (32.8) | |
| | 2016-2017 | 400 (23.9) | 350 (20.9) | |
| Site of Disease | Head | 602 (36.0) | 549 (32.8) | 0.07 |
| | Body & Neck | 375 (22.4) | 384 (23.0) | |
| | Tail | 315 (18.8) | 350 (20.9) | |
| | Unspecified | 380 (22.7) | 389 (23.3) | |
| Radiation | Yes | 69 (4.1) | 80 (4.8) | 0.03 |
| | No | 1,603 (95.9) | 1,592 (95.2) | |
| Time to Chemotherapy (mean (SD)) | Weeks | 4.5 (2.8) | 4.5 (2.9) | 0.008 |
| Charlson Comorbidity Index | 0 | 610 (36.5) | 632 (37.8) | 0.03 |
| | 1 | 515 (30.8) | 502 (30.0) | |
| | >1 | 547 (32.7) | 538 (32.2) | |
| ERCP | Yes | 399 (23.9) | 284 (17.0) | 0.17 |
| | No | 1,273 (76.1) | 1,388 (83) | |
| Infection | Any Location | 473 (28.3) | 323 (19.3) | 0.21 |
| | Intra-Abdominal | 97 (5.8) | 74 (4.4) | 0.06 |
| | Respiratory | 117 (7.0) | 88 (5.3) | 0.07 |
| | Genitourinary | 196 (11.7) | 133 (8.0) | 0.13 |
| | Blood | 109 (6.5) | 78 (4.7) | 0.08 |
| | Skin | 39 (2.3) | 35 (2.1) | 0.02 |
| | Other Bacterial | 72 (4.3) | 54 (3.2) | 0.06 |
| First-Line Chemotherapy | Gemcitabine * | 1409 (84.3) | 1409 (84.3) | <0.001 |
| | Fluorouracil * | 263 (15.7) | 263 (15.7) | |

© 2023 Fulop DJ et al. *JAMA Network Open.*

Summary statistics of baseline variables after performing propensity score (PS) matching. The mean and standard deviation (SD) are reported for continuous variables and *n* (%) for categorical variables. Abbreviations: SMD, standardized mean difference; ERCP, Endoscopic Retrograde Cholangiopancreatography; SES, socioeconomic status. * Chemotherapy type was not included in PS estimation.

© 2023 Fulop DJ et al. *JAMA Network Open.*

**eTable 6.** Result of Schoenfeld test in propensity-matched analyses

| | ***P*-value** |
|---|---|
| All Chemotherapy | 0.64 |
| Gemcitabine | 0.59 |
| Fluorouracil | 0.41 |

Schoenfeld residual *P*-values for the Cox proportional hazards regression models in the propensity-matched analyses.

© 2023 Fulop DJ et al. *JAMA Network Open.*

**eTable 7.** Sensitivity analysis using varying antibiotic receipt definitions

| Antibiotic Receipt Definitions | **PS-Adjusted HR (95% CI)** | ***P*-value** |
|---|---|---|
| ≥3 days | 0.89 (0.83-0.96) | 0.002 |
| ≥5 days | 0.89 (0.83-0.96) | 0.001 |
| ≥7 days | 0.88 (0.82-0.94) | <0.001 |
| ≥10 days | 0.86 (0.80-0.91) | <0.001 |
| ≥14 days | 0.86 (0.81-0.92) | <0.001 |

Hazard ratios for overall survival in sensitivity analysis using varying antibiotic receipt duration requirements. Abbreviations: PS, Propensity Score; HR, Hazard Ratio; CI, Confidence Interval.

© 2023 Fulop DJ et al. *JAMA Network Open.*

**eTable 8.** Sensitivity analysis for infection capture

| | **PS-Adjusted HR (95% CI)** | ***P*-value** |
|---|---|---|
| All Chemotherapy | 0.93 (0.87-0.99) | 0.04 |
| Gemcitabine | 0.92 (0.85-0.99) | 0.04 |
| FU | 1.01 (0.85-1.19) | 0.93 |

Hazard ratios for overall survival in sensitivity analysis including patients with blood infections but without documented antibiotic receipt in the antibiotic group. Abbreviations: OS, Overall Survival; PS, Propensity Score; HR, Hazard Ratio; CI, Confidence Interval.

© 2023 Fulop DJ et al. *JAMA Network Open.*

**eFigure 1.** Propensity score distribution before and after matching

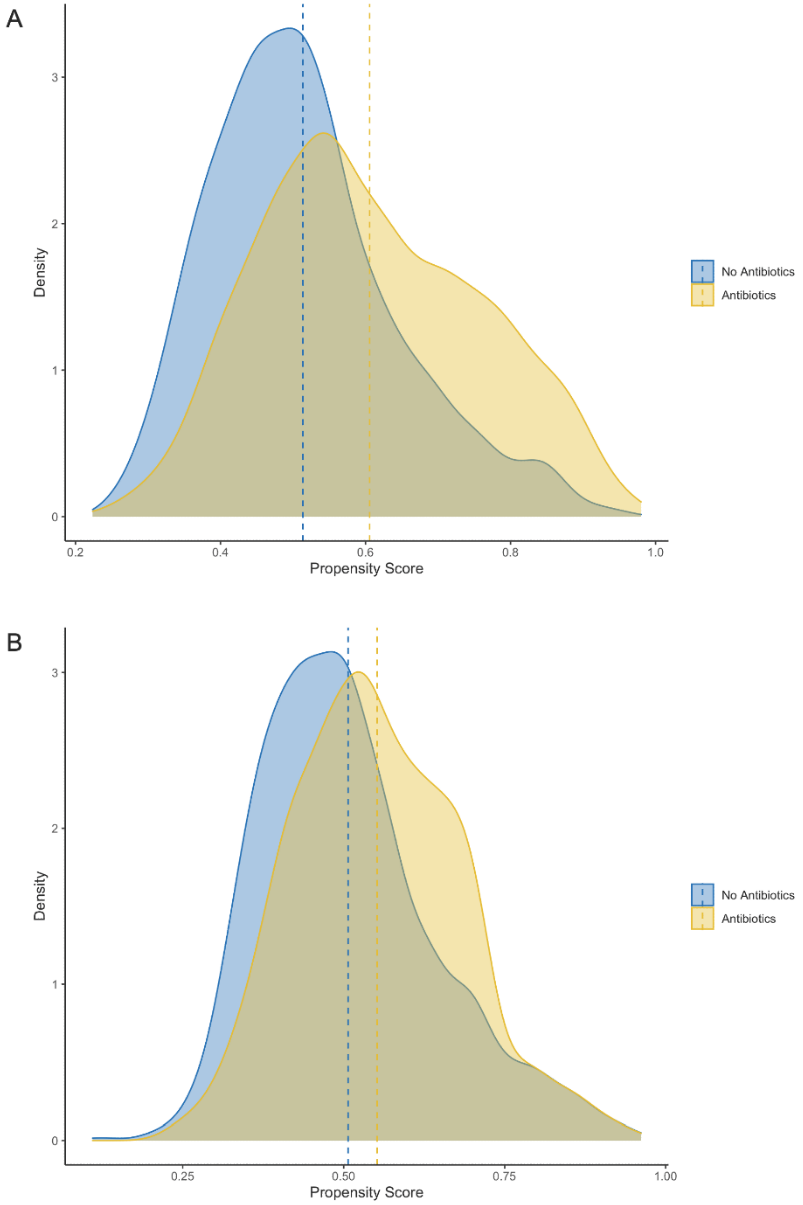


**Legend:** Distribution of propensity scores before (A) and after (B) matching for the patients who received antibiotics (yellow) and did not receive antibiotics (blue).

© 2023 Fulop DJ et al. *JAMA Network Open.*

**eFigure 2.** Kaplan-Meier curves in the unmatched cohort

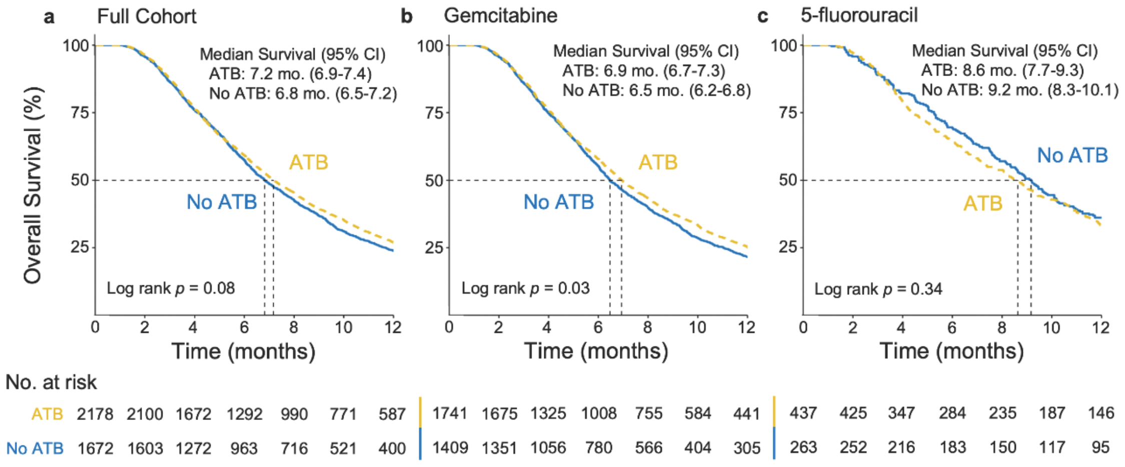


**Legend:** Kaplan-Meier survival curves for the full cohort prior to propensity score matching depicting 12-month survival for patients who received antibiotics in the two-month peri-chemotherapy exposure period (yellow, dashed) and patients who did not receive antibiotics (blue) in (a) the full unmatched cohort of 3,850 patients, as well as stratified by (b) gemcitabine, and (c) fluorouracil. Dashed lines (black) show median survival times for each group. Survival is calculated as months since diagnosis. Abbreviations: ATB, antibiotics; CI, confidence interval.

© 2023 Fulop DJ et al. *JAMA Network Open.*

## eReferences:

1. Enewold L, Parsons H, Zhao L, et al. Updated Overview of the SEER-Medicare Data: Enhanced Content and Applications. J Natl Cancer Inst Monogr. 2020;2020: 3-13.
2. Cancer Medications Enquiry Database (CanMED). Surveillance Research Program SEER website tool.: Division of Cancer Control and Population Sciences, National Cancer Institute., Version 1.10.3, 2021.
3. Fabricio S. P. Kury OB. Desiderata for Drug Classification Systems for their Use in Analyzing Large Drug Prescription Datasets.


© 2023 Fulop DJ et al. *JAMA Network Open.*
