# Supplementary material for: Association of Antibiotic Receipt With Survival Among Patients With Metastatic Pancreatic Ductal Adenocarcinoma Receiving Chemotherapy
Source: JAMA Netw Open. 2023 Mar 23;6(3):e234254. doi: 10.1001/jamanetworkopen.2023.4254 (PMC10037151; doi:10.1001/jamanetworkopen.2023.4254)
Supplement: Supplement 2. — Data Sharing Statement [file jamanetwopen-e234254-s002.pdf]

Data Sharing Statement

Fulop. Association of Antibiotic Receipt With Survival Among Patients With Metastatic Pancreatic Ductal Adenocarcinoma Receiving Chemotherapy. *JAMA Netw Open*. Published March 23, 2023. doi:10.1001/jamanetworkopen.2023.4254

Data
Data available: No
